# Neohesperidin Dihydrochalcone and Neohesperidin Dihydrochalcone-O-Glycoside Attenuate Subcutaneous Fat and Lipid Accumulation by Regulating PI3K/AKT/mTOR Pathway In Vivo and In Vitro

**DOI:** 10.3390/nu14051087

**Published:** 2022-03-04

**Authors:** Minseo Kwon, Yerin Kim, Jihye Lee, John A. Manthey, Yang Kim, Yuri Kim

**Affiliations:** 1 Department of Nutritional Science and Food Management, Ewha Womans University, Seoul 03760, Korea; minseu94@naver.com (M.K.); elle_rossy@naver.com (Y.K.); boboom38@naver.com (J.L.); 2 U.S. Horticultural Research Lab, U.S. Department of Agriculture, Agricultural Research Service, 2001 South Rock Road, Fort Pierce, FL 34945, USA; john.manthey@usda.gov; 3 Center for Food & Bioconvergence, Seoul National University, Seoul 08826, Korea; yankim@snu.ac.kr

**Keywords:** neohesperidin dihydrochalcone, glycoside, obesity, subcutaneous adipose tissue, lipogenesis, PI3K/AKT/mTOR

## Abstract

Neohesperidin dihydrochalcone (NHDC), a semi-natural compound from bitter orange, is an intense sweetener. The anti-obesity effects of NHDC and its glycosidic compound, NHDC-O-glycoside (GNHDC), were investigated. C57BLKS/J db/db mice were supplemented with NHDC or GNHDC (100 mg/kg b.w.) for 4 weeks. Body weight gain, subcutaneous tissues, and total adipose tissues (sum of perirenal, visceral, epididymal, and subcutaneous adipose tissue) were decreased in the NHDC and GNHDC groups. Fatty acid uptake, lipogenesis, and adipogenesis-related genes were decreased, whereas β-oxidation and fat browning-related genes were up-regulated in the sweetener groups. Furthermore, both sweeteners suppressed the level of triacylglycerol accumulation, lipogenesis, adipogenesis, and proinflammatory cytokines in the 3T3-L1 cells. The PI3K/AKT/mTOR pathway was also down-regulated, and AMP-acttvated protein kinase (AMPK) was phosphorylated in the treatment groups. These results suggest that NHDC and GNHDC inhibited subcutaneous fat and lipid accumulation by regulating the PI3K/AKT/mTOR pathway and AMPK-related lipogenesis and fat browning.

## 1. Introduction

Obesity is defined as excessive fat accumulation that causes serious health problems. The World Health Organization (WHO) has reported that obesity increased around three-fold worldwide from 1975 to 2016 [1].

For these reasons, WHO proposed obesity as a global epidemic in the 21st century at the WHO Consultation on Obesity [2]. Being obese is also associated with diabetes mellitus [3,4], cardiovascular diseases [5,6], and cancers [4], all associated with metabolic disorders. There are several fat accumulation-related mechanisms, including lipogenesis or adipogenesis [7]. On the contrary, β-oxidation [8] and fat browning [9,10] are inhibitory mechanisms of fat accumulation.

Sugar consumption has dramatically increased since the mid-1960s [11,12], and sugar-sweetened beverages are considered to be the main driving factor [13]. The WHO has strongly recommended reducing sugar intake levels to less than 10% of the total energy intake [14]. Previous studies have shown that increased sugar consumption could enhance the prevalence of obesity in children and adults [15,16].

Alternative sweeteners have been suggested as a solution, in order to reduce sugar consumption. Sweeteners are classified into bulk sweeteners and intense sweeteners [17]. They are divided into these two categories depending on their calorie content. Bulk sweeteners produce calories, whereas intense sweeteners generate no or only minimal caloric value. Intense sweeteners also possess higher relative sweetness than sucrose [18]. Thus, their use is expected to prevent obesity or type 2 diabetes mellitus by using alternative sweeteners as a substitute for sugar.

Neohesperidin dihydrochalcone (NHDC) is an intense sweetener. It is extracted and processed from neohesperidin, its parent flavanone [19]. Flavanone glycosides are mainly found in the peels of oranges, and the dihydrochalcone form is synthesized through hydrogenation [20]. NHDC is a semi-natural compound manufactured from neohesperidin, a naturally occurring flavonoid obtained from bitter orange, *Citrus aurantium* [19,21], and it possesses high solubility and stability. The relative sweetness of NHDC is 250–2000 times higher than a sucrose solution [22,23]. In addition, NHDC can be utilized for masking the bitterness of other compounds [24]. It has been reported that this functional alternative sweetener also exerts antioxidant or anti-inflammatory effects [25,26].

Glycoside is a compound with one or more sugars linked by a glycosidic bond to non-sugar molecules [27]. There are four types of glycoside according to the location of the linkage: O-, C-, N-, and S-glycoside. Glycosides are known to exert anti-obesity [28] or anti-diabetic effects [29]. Some glycosidic compounds prevent diabetes by targeting the sodium-dependent glucose cotransporter (SGLT), dipeptidyl peptidase IV (DPP-IV), glucagon-like peptide 1 (GLP1), and peroxisome proliferator-activated receptor gamma (PPARγ) [30]. Previous studies reported that NHDC had a radical scavenging activity and inhibited the reactive oxygen species (ROS) [31] as well as exerted anti-inflammatory effects in a paraquat-induced acute liver injury model [26]. Although the anti-obesity effect of NHDC was studied in vitro [25], the effect of NHDC and its glycosides on adipose tissues and their mechanisms remain to be identified. Thus, in the present study, the decreased subcutaneous adipose tissue effects of both NHDC, its glycoside compound, GNHDC, as well as their molecular mechanisms were investigated both in vivo and in vitro.

## 2. Materials and Methods

### 2.1. Materials

**Figure 1:**
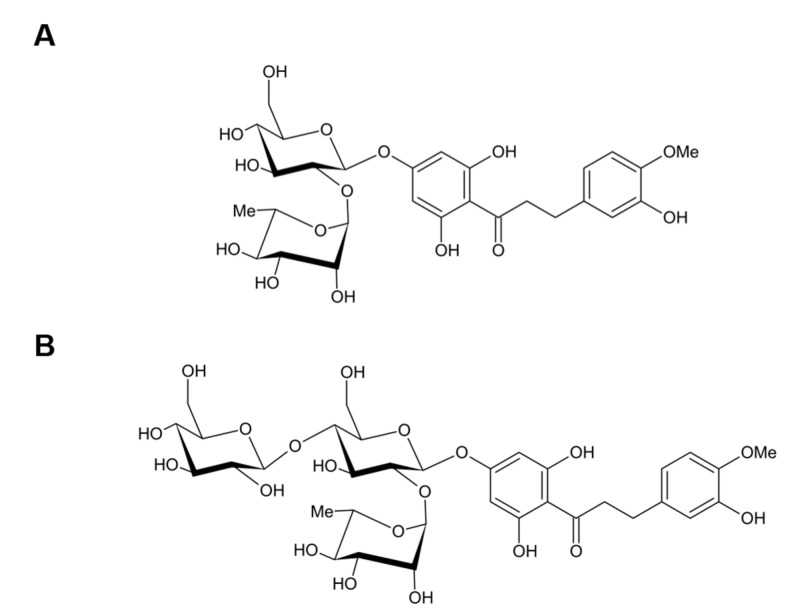
Structure of NHDC and GNHDC. The molecular structure of (**A**) NHDC and (**B**) GNHDC are presented. NHDC, neohesperidin dihydrochalcone; GNHDC, NHDC-O-glycoside.

NHDC (1-(4-((2-O-[6-Deoxy-α-L-mannopyranosyl]-β-D-glucopyranosyl)oxy)-2,6-dihydroxyphenyl)-3-[3-hydroxy-4-methoxyphenyl]-1-propanone), and NHDC-O-glycoside (1-(4-((2-O-[6-Deoxy-α-L-mannopyranosyl]-(4-O-α-D-glucopyranosyl]-β-D-glucopyranosyl)oxy)-2,6-dihydroxyphenyl)-3-[3-hydroxy-4-methoxyphenyl]-1-propanone) (GNHDC) were kindly provided by Dr. Manthey from the U.S. Horticultural Research Laboratory, Agricultural Research Service, U.S. Department of Agriculture (Figure 1). These chemicals were diluted to 30, 50, and 100 μM NHDC in 0.2% dimethyl sulfoxide (DMSO) (Sigma-Aldrich, St. Louis, MO, USA) or GNHDC in 0.1% ethanol (Merck, Darmstadt, Germany) in a culture medium for the in vitro study. Both chemicals were dissolved in phosphate-buffered saline (PBS) for the in vivo study.

### 2.2. Animal Studies

Five-week-old male C57BLKS/J db/db mice were purchased from the Central Lab Animal Inc. (Seoul, Korea). A leptin receptor-deficient (db/db) mice model was chosen to assess the effects of blood glucose levels in the present study. Therefore, C57BLKS/J db/db mice were selected to be the experimental animal model since this model has often been used for type 2 diabetes mellitus or metabolic disorder-related studies [32,33]. All mice were maintained individually at 22 ± 2 °C, 50 ± 5% humidity, and 12 h/12 h light/dark cycles. The animals were randomly divided into three groups (*n* = 9 mice per group) based on both body weight and fasting blood glucose levels after an acclimation period of 2 weeks, as follows: animals fed PBS (Ctrl), animals fed 100 mg/kg body weight (b.w.) NHDC (NHDC), and animals fed 100 mg/kg b.w. GNHDC (GNHDC). All reagents were given as an oral gavage 5 days per week, for 4 weeks. They were fed a modified American Institute of Nutrition (AIN)-93G diet (Raonbio, Yongin, Korea) and water ad libitum. The composition of the AIN-93G diet is presented in Table 1. During the experimental period, body weight, food intake, and water intake were recorded twice a week. Fasting blood glucose levels were measured from the tail vein by using Accu-Check (Roche, Mannheim, Germany). The mice were fasted 12 h before euthanasia and anesthetized through isoflurane inhalation for 1–2 min [34]. After the mice were sacrificed, all the blood was centrifuged at 13,000× *g*, at 4 °C, for 15 min. For adipose tissue dissection, we followed the protocol by Bagchi and MacDougald, 2019 [35]. For subcutaneous fat isolation, anterior subcutaneous adipose tissues located between the scapulae and posterior subcutaneous adipose tissues comprised of the dorsolumbar, inguinal, and gluteal parts were isolated [35]. Perirenal, visceral, and epididymal adipose tissues were also collected. Adipose tissues were rinsed with PBS and then frozen in liquid nitrogen. All samples were stored at −80 °C until further analysis. The study procedures and experiments were approved by the Institutional Animal Care and Use Committee of Ewha Womans University (IACUC approval number: 19-052).

### 2.3. Oral Glucose Tolerance Test

An oral glucose tolerance test (OGTT) was performed after 2 weeks by providing a glucose (1 g/kg b.w.) solution to overnight fasting mice. Their blood glucose levels were monitored at 0, 30, 60, 90, and 120 min using Accu-Check (Roche). The area under the curve (AUC) of the OGTT was calculated from the OGTT curves.

### 2.4. Biochemical Analysis

Levels of total cholesterol, high density lipoprotein (HDL)-cholesterol, and triacylglycerols were measured by commercial kits (Asan Pharmaceutical, Seoul, Korea). Low density lipoprotein (LDL) cholesterol was calculated using the Friedewald equation: LDL cholesterol = Total cholesterol-HDL-cholesterol-(triacylglycerols/5). Leptin and adiponectin were measured with an enzyme-linked immunosorbent assay (ELISA) kit (Crystal Chem, Elk Grove Village, IL, USA). Non-esterified fatty acid was measured using an enzymatic colorimetric method assay (FUJIFILM Wako Pure Chemical Corporation, Osaka, Japan). Insulin was also measured using an ELISA kit (FUJIFILM Wako Pure Chemical Corporation). The degree of insulin resistance was calculated with the HOMA-IR, Homeostatic Model Assessment for Insulin Resistance index [36].

### 2.5. Histological Analysis

Subcutaneous adipose tissues were fixed with 10% formaldehyde and embedded in paraffin. Hematoxylin and eosin (H&E) staining was performed after deparaffinizing with xylene and rehydrating. Two serial tissue sections, 5 μm thick, were cut on a microtome from each paraffin-embedded specimen. Stained sections were observed with a microscope (Nikon, Tokyo, Japan) and captured. Adipocyte areas of subcutaneous adipose tissues were quantified using ImageJ (National Institutes of Health, Bethesda, MD, USA).

### 2.6. Quantitative Real-Time PCR

Samples were extracted using Trizol reagent (Invitrogen, Carlsbad, CA, USA). The concentration and quality of the RNA were checked by Nanodrop (Thermo Scientific, Waltham, MA, USA), and cDNA was produced with RevertAid reverse transcriptase (Thermo Scientific) at 42 °C for 1 h, followed by 72 °C for 3 min. Quantitative real-time PCR was performed using Rotor-Gene^®^ Q (Qiagen, Hilden, Germany) and 2X SYBR Green PCR master mix (Qiagen), following the manufacturer’s protocol. Glyceraldehyde 3-phosphate dehydrogenase (Gapdh) was used as an internal control for both in vivo and in vitro studies. The primer sequences used are presented in Table 2.

### 2.7. Cell Culture

A murine preadipocyte cell line, 3T3-L1, was purchased from the American Type Culture Collection (ATCC, Manassas, VA, USA). The cells were maintained in Dulbecco’s modified Eagle’s medium (DMEM) (Welgene, Gyeongsan, Korea), supplemented with 10% bovine calf serum (Gibco, Grand Island, NY, USA) and 1% penicillin/streptomycin (P/S) (Invitrogen) in a 37 °C and 5% CO_2_ environment. The cells were differentiated into adipocytes in DMEM, 10% fetal bovine serum (FBS) (Gibco), 1% P/S, 500 μM isobutylmethylxanthine (Sigma-Aldrich), 1 μM dexamethasone (Sigma-Aldrich), and 10 μg/mL insulin (Welgene) for 2 days. After the differentiation, the adipocytes were incubated in 10% FBS, 1% P/S, and 1 μg/mL insulin for 6 days. The cells were treated with 30, 50, or 100 μM NHDC or GNHDC for 8 days.

### 2.8. Oil Red O Staining

Triacylglycerol accumulation was measured by oil red O staining (Sigma-Aldrich). Differentiated adipocytes were rinsed with PBS and fixed with 10% formaldehyde. The cells were stained with an oil red O staining solution after being washed with 60% isopropyl alcohol. The staining solution was washed away with distilled water, and the slides were subsequently dried. The stained lipid droplets were captured by an Eclipse TS100 (Nikon) before the staining solution was dissolved in 100% isopropyl alcohol. Then, for quantification, the absorbance was recorded with a microplate reader (Molecular Device, Sunnyvale, CA, USA) at 500 nm.

### 2.9. Cell Viability Assay

The cell viability was measured with a 3-(4,5-dimethylthiazol-2-yl)-2,5-diphenyl tetrazolium bromide (MTT) (Sigma-Aldrich) assay. The 3T3-L1 cells were seeded into 96-well plates and treated with 50 μM NHDC or GNHDC for 8 days. The culture medium was removed, and the cells were treated with a medium containing 500 μg/mL MTT solution under 37 °C and 5% CO_2_ for 3 h. Then, the absorbance was recorded using a microplate reader (Molecular Device) at 560 nm.

### 2.10. Western Blotting

The protein concentration of the differentiated adipocytes was measured with a Bradford protein assay using a Bio-Rad Protein Assay Kit (Bio-Rad, Hercules, CA, USA). The samples were denatured and separated by electrophoresis using sodium dodecyl sulphate-polyacrylamide gel electrophoresis (SDS-PAGE). Then, the protein samples were transferred to polyvinylidene fluoride membranes (Millipore, Billerica, MA, USA) and blocked with 5% bovine serum albumin or skim milk in Tris-buffered saline containing Tween 20 (TBS-T). The membranes were incubated at 4 °C overnight with primary antibodies directed against the following proteins: phosphoinositide 3 kinase (PI3K), phospho-phosphoinositide 3 kinase (p-PI3K), protein kinase B (AKT), phospho-protein kinase B (p-AKT), mammalian target of rapamycin (mTOR), phospho-mammalian target of rapamycin (p-mTOR), AMP-activated protein kinase (AMPK), and phospho-AMP-activated protein kinase (p-AMPK) (Cell Signaling Technology, Danvers, MA, USA), with β-actin (Abcam, Cambridge, UK) as the loading control. The membranes were rinsed with TBS-T and then incubated with the corresponding secondary goat anti-mouse or anti-rabbit antibodies for 1 h. The protein bands were detected by an enhanced chemiluminescence (ECL) reagent (Animal Genetics Inc., Suwon, Korea).

### 2.11. Statistical Analysis

All results are presented as the mean ± standard error of the mean (SEM) and were analyzed by one-way ANOVA with a Newman–Keuls post hoc test or an unpaired Student’s *t*-test. All in vitro experiments were performed at least three times. *p*-values less than 0.05 were considered significant. GraphPad Prism (GraphPad Software Inc., San Diego, CA, USA) software was used for evaluating the significance of the data. This statistical analysis is for both in vivo and in vitro studies.

## 3. Results

### 3.1. Effects of NHDC and GNHDC on Body Weight, Tissue Weight, Lipid Profile, and Cytokines in db/db Mice

After 4 weeks of supplementation, the body weight and adipose tissue weight of the mice were measured (Table 3). Their final body weight tended to be reduced in both the NHDC and GNHDC supplement groups, but the change was not significant (*p* > 0.05). The body weight gain in the GNHDC group significantly decreased by 29.1% (*p* < 0.05) as compared with the Ctrl group. The subcutaneous adipose tissue weight significantly decreased by 48.4% in the NHDC group (*p* < 0.05) and by 39.6% in the GNHDC group (*p* < 0.05) as compared with the Ctrl group. The visceral adipose tissue weight was not significantly different between groups (*p* > 0.05). The total adipose tissue weight—sum of the perirenal, visceral, epididymal, and subcutaneous adipose tissues—was also significantly decreased by 21.8% in the NHDC group (*p* < 0.05) and by 19.2% in the GNHDC group (*p* < 0.05).

Total cholesterol, LDL-cholesterol, HDL-cholesterol, triacylglycerols, and non-esterified fatty acid (NEFA) were analyzed to evaluate the effects of NHDC and GNHDC on the plasma lipid profile (Table 3). The total cholesterol significantly decreased by 8.3% in the GNHDC group (*p* < 0.05) as compared to the Ctrl group. Although the levels of LDL-cholesterol and triacylglycerols tended to be reduced and the levels of HDL-cholesterol tended to be slightly increased in both the NHDC and GNHDC supplemented groups, these changes were not significant (*p* > 0.05). NEFA significantly decreased by 16.3% in the GNHDC group (*p* < 0.05) as compared to the Ctrl group. In addition, there was a tendency of decreasing leptin and increasing adiponectin in the GNHDC group; however, they were not significant (*p* > 0.05). Insulin was significantly increased in the NHDC group (*p* < 0.01), and it decreased slightly in the GNHDC group as compared to the Ctrl group (*p* > 0.05). There were no significant differences in HOMA-IR levels between the groups (*p* > 0.05).

### 3.2. Effects of NHDC and GNHDC on Body Weight, Food Intake, Water Intake, Fasting Blood Glucose Levels, and OGTT in db/db Mice

**Figure 2:**
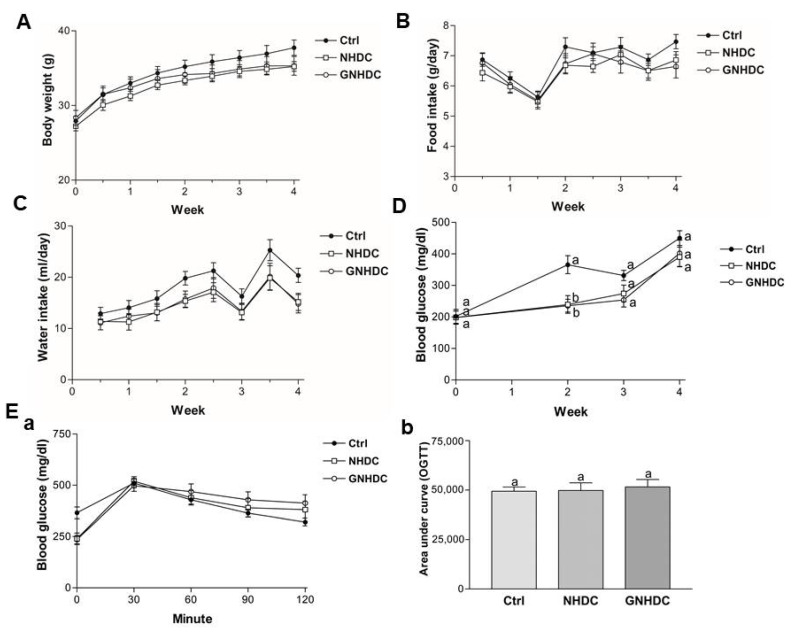
Effects of NHDC and GNHDC on body weight, food intake, water intake, fasting blood glucose levels, and OGTT in db/db mice. Body weight, food intake, water intake, fasting blood glucose levels, and OGTT were analyzed in db/db mice. (**A**) Body weight, (**B**) Food intake, and (**C**) Water intake, and (**D**) changes in fasting blood glucose levels during the experimental period. (**E**) (**a**) OGTT and (**b**) AUC for OGTT are presented. All data are shown as the mean ± SEM (*n* = 9 per group) and were analyzed by one-way ANOVA with a Newman–Keuls post hoc test. OGTT, oral glucose tolerance test; AUC, area under the curve; Ctrl, db/db mice control; NHDC, db/db mice with neohesperidin dihydrochalcone supplement; GNHDC, db/db mice with NHDC-O-glycoside supplement. The same letter indicates no significant differences (*p* > 0.05) and different letters indicate significant differences (*p* < 0.05, ANOVA).

Body weight, food intake, and water intake were measured during the whole experimental period (Figure 2A–C), and they showed no significant differences between groups in every time point. Fasting blood glucose levels were monitored to evaluate the effects of NHDC and GNHDC supplementation on hyperglycemia in db/db mice (Figure 2D). Compared to the Ctrl group, levels of fasting blood glucose were significantly decreased by both NHDC (34.5%, *p* < 0.01) and GNHDC (35.5%, *p* < 0.01) after 2 weeks of supplementation. However, this effect was reduced after 3 weeks. In addition, there was no significant difference in glucose tolerance among all groups, and the AUC for OGTT was not significantly different between the Ctrl and sweetener groups (all *p* > 0.05) (Figure 2E).

### 3.3. Effects of NHDC and GNHDC on the Subcutaneous Adipocyte Area and the Gene Expressions for Lipid Metabolism in Subcutaneous Adipose Tissues

**Figure 3:**
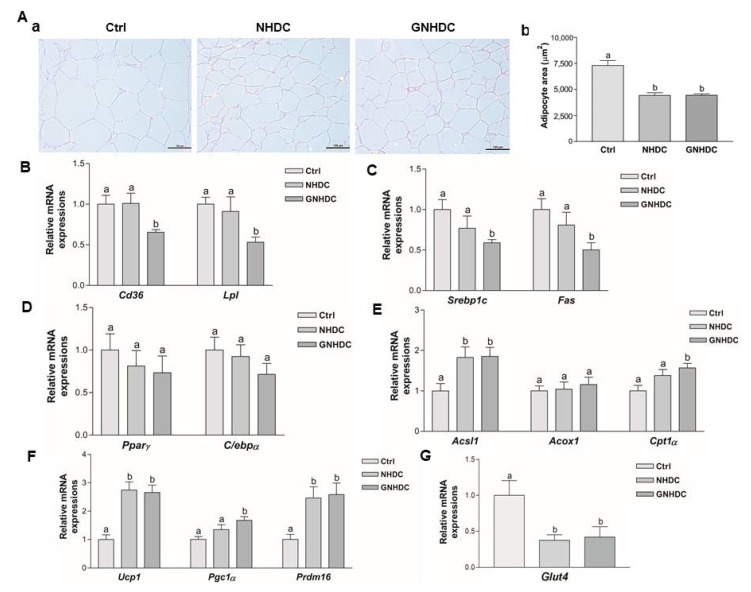
Effects of NHDC and GNHDC on the subcutaneous adipocyte area and the gene expressions for lipid metabolism in subcutaneous adipose tissues. Subcutaneous adipose tissues were analyzed by (**A**) (**a**) H&E staining (magnification 200×, scale bar 10 μm) with Ctrl, NHDC, and GNHDC. (**b**) Adipocyte area of subcutaneous adipose tissues was quantified (*n* = 3 per group). Expression levels of mRNA were analyzed for (**B**) fatty acid uptake-related genes, Cd36 and Lpl, (**C**) lipogenesis-related genes, *Srebp1c* and *Fas*, (**D**) adipogenesis-related genes, *Pparγ* and *C/ebpα*, (**E**) β-oxidation-related genes, *Acsl1*, *Acox1*, and *Cpt1α*, (**F**) fat browning-related genes, *Ucp1*, *Pgc1α*, and *Prdm16*, and (**G**) the insulin-responsive glucose transporter gene, *Glut4*. All data (**B**–**G**) are shown as the mean ± SEM quantified (*n* = 9 per group) and were analyzed by one-way ANOVA with a Newman–Keuls post hoc test. *Acox1*, acyl-CoA oxidase 1; *Acsl1*, acyl-CoA synthetase long-chain family member 1; *Cd36*, cluster of differentiation 36; *C/ebpα*, CCAAT/enhancer binding protein alpha; *Cpt1α*, carnitine palmitoyltransferase 1 alpha; *Fas*, fatty acid synthase; H&E, hematoxylin and eosin; *Lpl*, lipoprotein lipase; *Pgc1α*, peroxisome proliferator-activated receptor gamma coactivator 1 alpha; *Pparγ*, peroxisome proliferator-activated receptor gamma; *Prdm16*, positive regulatory domain 16; *Srebp1c*, sterol regulatory element-binding protein 1; *Ucp1*, uncoupling protein 1; *Glut4*, glucose transporter type 4; Ctrl, db/db mice control; NHDC, db/db mice with neohesperidin dihydrochalcone supplement; GNHDC, db/db mice with NHDC-O-glycoside supplement. The same letter indicates no significant differences (*p* > 0.05) and different letters indicate significant differences (*p* < 0.05, ANOVA).

The histological changes in the subcutaneous adipose tissues were observed by H&E staining, and the adipocyte area was measured to evaluate the effects of NHDC and GNHDC supplementation on this fat tissue (Figure 3A). The size of the lipid droplets was quantified, and they were significantly reduced by 39.4% in the NHDC (*p* < 0.01) and 39.2% in the GNHDC group (*p* < 0.01) as compared with the Ctrl group.

To evaluate the effects of NHDC and GNHDC on fatty acid uptake, lipogenesis, adipogenesis, β-oxidation, and fat browning-related genes, quantitative real-time PCR was performed. The mRNA expression levels of *Cd36* and *Lpl* were analyzed for fatty acid uptake (Figure 3B). The mRNA expressions of *Cd36* and *Lpl* were down-regulated by 34.6% and 46.8%, respectively, in the GNHDC group (*p* < 0.05 for both) relative to the Ctrl group. *Srebp1c* and *Fas* for lipogenesis-related genes (Figure 3C) and *Pparγ* and *C/ebpα* for adipogenesis-related genes were analyzed (Figure 3D). Expressions of *Srebp1c* and *Fas* were decreased by 41.0% and 49.7%, respectively, in the GNHDC group (*p* < 0.05 for both) as compared to the Ctrl group. Expressions of *Pparγ* and *C/ebpα* tended to be decreased by the NHDC and GNHDC supplementation, but the change was not statistically significant (*p* > 0.05).

The mRNA expression levels of the β-oxidation-related *Acsl1*, *Acox1*, and *Cpt1α* genes were analyzed (Figure 3E). Expression of *Acsl1* was up-regulated about 1.8-fold in the NHDC and GNHDC groups (*p* < 0.05 for both) as compared to the Ctrl group. Expression of *Acox1* tended to be increased by both supplements, but it was not statistically significant (*p* > 0.05). Expression of *Cpt1α* was up-regulated about 1.5-fold in the GNHDC group (*p* < 0.05).

The mRNA expression of the fat browning-related *Ucp1*, *Pgc1α*, and *Prdm16* genes were analyzed (Figure 3F). Expression of *Ucp1* was up-regulated in the NHDC and GNHDC group (*p* < 0.01 for both) as compared to the Ctrl group. The expression of *Pgc1α* was up-regulated about 1.6-fold in the GNHDC group (*p* < 0.01). The expression of *Prdm16* was up-regulated about 2.4-fold in the NHDC and GNHDC groups (*p* < 0.01 for both), respectively. The mRNA expression of the *Glut4* gene was analyzed. Expression of *Glut4* was down-regulated by about 62.59% in the NHDC and 58.05% in the GNHDC group (*p* < 0.05 for both) compared to the Ctrl group (Figure 3G).

### 3.4. Effects of NHDC and GNHDC on Lipid Accumulation and Cell Viability in 3T3-L1 Cells

**Figure 4:**
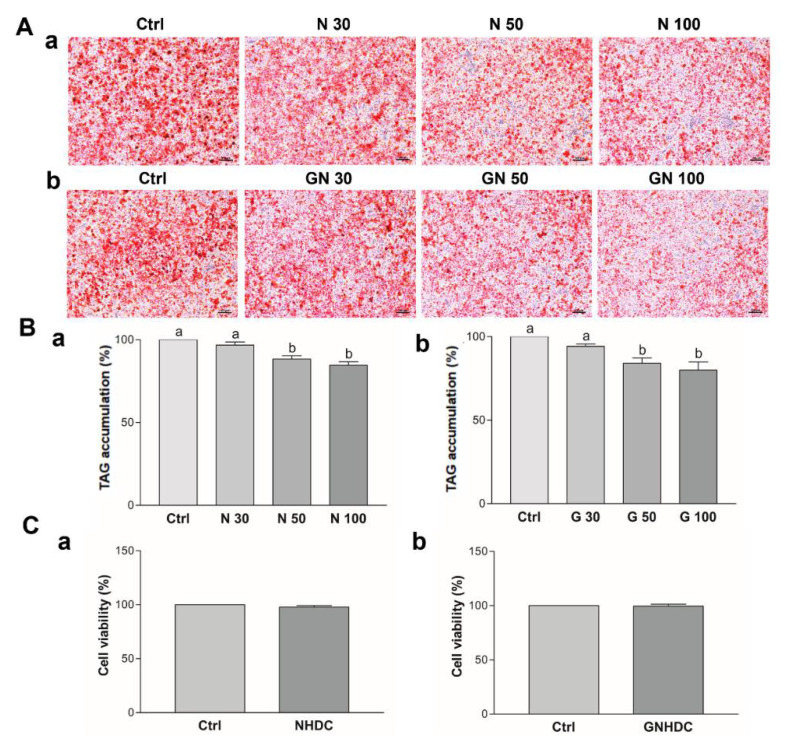
Effects of (**a**) NHDC and (**b**) GNHDC on lipid accumulation and cell viability in 3T3-L1 cells. Lipid accumulation was measured by (**A**) oil red O staining (magnification 100×, scale bar 100 μm) and (**B**) Triacylglycerol accumulation was quantified with Ctrl, 30 μM, 50 μM, and 100 μM (**a**) NHDC or (**b**) GNHDC. (**C**) Cell viability in 3T3-L1 cells was evaluated with 50 μM (**a**) NHDC or (**b**) GNHDC for 8 days by MTT assay. All data are shown as the mean ± SEM and were analyzed by one-way ANOVA with a Newman–Keuls post hoc test or an unpaired Student’s *t*-test (*n* = 3). TAG, triacylglycerols; Ctrl, control; NHDC, neohesperidin dihydrochalcone; N 30, 30 μM NHDC; N 50, 50 μM NHDC; N 100, 100 μM NHDC; GNHDC, NHDC-O-glycoside; GN 30, 30 μM GNHDC; GN 50, 50 μM GNHDC; GN 100, 100 μM GNHDC. The same letter indicates no significant differences (*p* > 0.05) and different letters indicate significant differences (*p* < 0.05, ANOVA).

After observing the significant decrease in adipose tissue weight in the NHDC and GNHDC groups in the animal study, 3T3-L1 preadipocytes were used to clarify the mechanism. To evaluate the effects of NHDC and GNHDC on triacylglycerols accumulation, oil red O staining was performed (Figure 4A). NHDC and GNHDC treatment decreased lipid droplet staining as compared to each Ctrl group (Figure 4B). It was decreased by 11.7% with 50 μM NHDC (*p* < 0.01), 15.4% with 100 μM NHDC (*p* < 0.01), 16.0% with 50 μM GNHDC (*p* < 0.05), and 20.0% with 100 μM GNHDC (*p* < 0.01) as compared to each Ctrl group, respectively. Based on the oil red O staining results, the lowest effective dose was 50 μM in both sweetener groups. To evaluate the cell toxicity of 50 μM NHDC and GNHDC, MTT assays were performed (Figure 4C). There was no significant difference in cell viability for both sweetener treatments. Therefore, further in vitro experiments were performed with 50 μM NHDC and GNHDC.

### 3.5. Effects of NHDC and GNHDC on Lipogenesis, Adipogenesis, and Proinflammatory Cytokines in 3T3-L1 Cells

**Figure 5:**
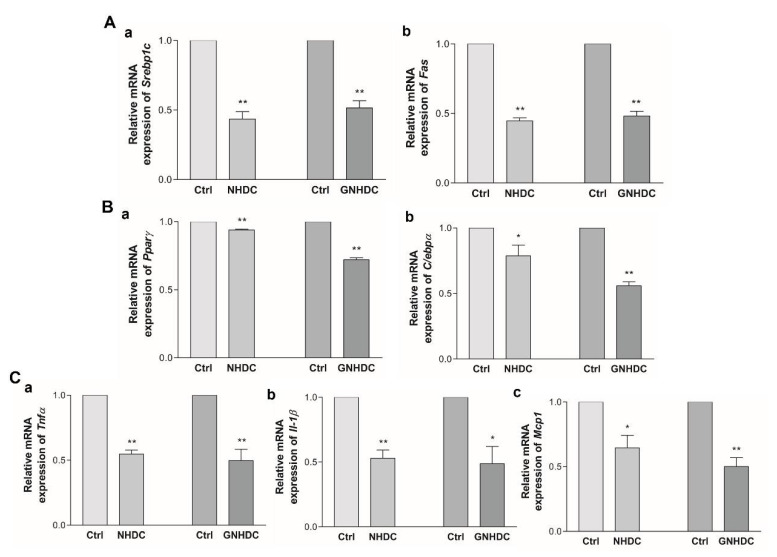
Effects of NHDC and GNHDC on lipogenesis, adipogenesis, and proinflammatory cytokines in 3T3-L1 cells. Expression of mRNA was analyzed for (**A**) lipogenesis-related genes, (**a**) *Srebp1c* and (**b**) *Fas*, (**B**) adipogenesis-related genes, (**a**) *Pparγ* and (**b**) *C/ebpα*, (**C**) proinflammatory cytokines-related genes, (**a**) *Tnfα*, (**b**) *Il-1β*, and (**c**) *Mcp1*. All data are shown as the mean ± SEM and were analyzed by an unpaired Student’s *t*-test (*n* = 3–4). Each asterisk indicates significant difference as compared to Ctrl; * (*p* < 0.05) and ** (*p* < 0.01). *C/ebpα*, CCAAT/enhancer binding protein alpha; *Fas*, fatty acid synthase; *Il-1β*, interleukin 1 beta; *Mcp1*, monocyte chemoattractant protein 1; *Pparγ*, peroxisome proliferator-activated receptor gamma coactivator 1 alpha; *Srebp1c*, sterol regulatory element-binding protein 1; *Tnfα*, tumor necrosis factor alpha; Ctrl, control; NHDC, neohesperidin dihydrochalcone; GNHDC, NHDC-O-glycoside.

To evaluate the effects of NHDC and GNHDC on lipogenesis and adipogenesis, quantitative real-time PCR was performed. The mRNA expressions of *Srebp1c* and *Fas* for lipogenesis (Figure 5A) and *Pparγ* and *C/ebpα* for adipogenesis (Figure 5B) were analyzed. The expression of *Srebp1c* decreased by 56.5% with NHDC (*p* < 0.01) and by 48.5% with the GNHDC treatment (*p* < 0.01) as compared to each Ctrl group. The expression of *Fas* decreased by 55.3% with NHDC (*p* < 0.01) and by 51.8% with the GNHDC treatment (*p* < 0.01) as compared to each Ctrl group. The mRNA expression of *Pparγ* decreased by 6.2% with NHDC (*p* < 0.01) and by 27.9% with the GNHDC treatment (*p* < 0.01) as compared to each Ctrl group. The mRNA expression of *C/ebpα* decreased by 21.2% with NHDC (*p* < 0.05) and by 44.2% with the GNHDC treatment (*p* < 0.01) as compared to each Ctrl group.

Next, the effects of both sweeteners on proinflammatory cytokines, including *Tnfα*, *Il-1β*, and *Mcp1,* were evaluated (Figure 5C). The mRNA expression of *Tnfα* decreased by 45.4% with NHDC (*p* < 0.01) and by 50.3% with the GNHDC treatment (*p* < 0.01) as compared to each Ctrl group. The expression of *Il-1β* decreased by 47.0% with NHDC (*p* < 0.01) and by 51.3% with the GNHDC treatment (*p* < 0.05) as compared to each Ctrl group. The expression of *Mcp1* decreased by 35.5% with NHDC (*p* < 0.05) and by 49.9% with the GNHDC (*p* < 0.01) treatment.

### 3.6. Effects of NHDC and GNHDC on the PI3K/AKT/mTOR Pathway and AMPK in 3T3-L1 Cells

**Figure 6:**
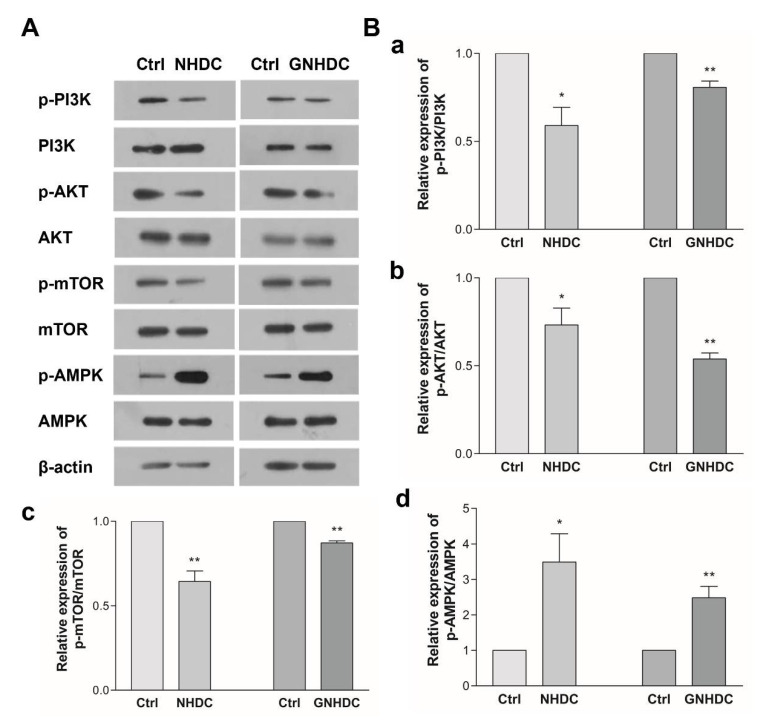
Effects of NHDC and GNHDC on the PI3K/AKT/mTOR pathway and AMPK in 3T3-L1 cells. The expression of protein was analyzed for the PI3K/AKT/mTOR pathway and AMPK. (**A**) Representative blots are presented; (**B**) Quantification of expression of (**a**) p-PI3K/PI3K, (**b**) p-AKT/AKT, (**c**) p-mTOR/mTOR, and (**d**) p-AMPK/AMPK. All data are shown as the mean ± SEM and were analyzed by an unpaired Student’s *t*-test (*n* = 3). Each asterisk indicates significant difference as compared to Ctrl; * (*p* < 0.05) and ** (*p* < 0.01). AKT, protein kinase B; AMPK, AMP-activated protein kinase; mTOR, mammalian target of rapamycin; PI3K, phosphoinositide 3 kinase; p-AKT, phospho-protein kinase B; p-AMPK, phospho-AMP-activated protein kinase; p-mTOR, phospho-mammalian target of rapamycin; p-PI3K, phospho-phosphoinositide 3 kinase; Ctrl, control; NHDC, neohesperidin dihydrochalcone; GNHDC, NHDC-O-glycoside.

To evaluate the effects of NHDC and GNHDC on the PI3K/AKT/mTOR pathway, western blot analysis was performed (Figure 6). In the present study, the expression of p-PI3K/PI3K decreased by 41.0% with NHDC (*p* < 0.05) and by 19.3% with the GNHDC treatment (*p* < 0.01) as compared to each Ctrl group. The expression of p-AKT/AKT decreased by 26.8% with NHDC (*p* < 0.05) and by 46.1% with the GNHDC treatment (*p* < 0.01). The expression of p-mTOR/mTOR decreased by 35.6% with NHDC (*p* < 0.01) and by 12.7% with the GNHDC treatment (*p* < 0.01). In the present study, expression of p-AMPK/AMPK increased by about 349.3% with NHDC (*p* < 0.05) and by 248.3% with the GNHDC treatment (*p* < 0.01).

## 4. Discussion

We selected the treatment dose based on previous studies and preliminary data. Previously, NHDC was used at 100–200 mg/kg b.w. [26,37,38,39] and no observed effect level (NOAEL) of NHDC was reported for 750 mg/kg b.w. in rats [40]. The Scientific Committee for Food of the European Community allocated NOAEL of 500 mg/kg b.w. and declared 5 mg/kg b.w. as an acceptable daily intake (ADI) level for NHDC [41]. NHDC is a semi-natural sweetener since it is naturally presented as neohesperidin, its parent flavone, before it is processed to NHDC [19]. NHDC is used as an ingredient in numerous processed foods, including drinks [19]. More than 90% of 14C-labeled NHDC was found to be excreted in the urine in a rat study due to its low rate of metabolism. Due to the low bioavailability of NHDC, it was suggested to provide not more than 1/1000 calories compared with an equivalent amount of sucrose [24].

Levels of water intake were decreased in both the NHDC and GNHDC groups, which suggested the anti-diabetic effects of these two sweeteners by alleviating polydipsia, a primary symptom of diabetes mellitus [42]. Previous studies have described the effects of alternative sweeteners on blood glucose levels [43,44]. Consistently, blood glucose levels were significantly decreased in supplemented groups in the second week of the experiment, whereas significant differences were not seen after the third week in the present study. Plasma insulin was unexpectedly higher in the NHDC group than in the Ctrl group. Insulin levels could be increased due to insulin resistance or a compensation for increased blood glucose in the sweetener groups. These results suggest that both sweeteners exhibit anti-diabetic effects in early diabetes. Glycosides are known for their anti-diabetic effects in several studies [45,46]. Several glycosidic substances suppress glucose reabsorption by inhibiting a normal transport of glucose via SGLT [45]. SGLT allows glucose to be transported into cells [47]. Conversely, SGLT1 and SGLT2 inhibitors prevent the reabsorption of glucose in the kidney or gut. Previous studies reported that blood glucose levels were lowered by SGLT1/SGLT2 inhibitors [48,49].

Although NHDC and GNHDC showed insignificant effects on regulating blood glucose levels at the end of the experiment, both sweeteners tended to decrease the body weight gain in db/db mice. The calories provided by the supplements used in this study were negligible considering the characteristic of intense sweeteners, which produce no or low calories [50]. NHDC produces about 2 kcal/g [17]. Food intake levels were not significantly changed in both sweetener groups as compared to the Ctrl group. This result suggests that the decreased body weight gain caused by these sweeteners was not due to decreased food intake.

All lipogenesis, adipogenesis, and proinflammatory cytokine-related genes were significantly down-regulated by NHDC or GNHDC treatment. Subcutaneous adipose tissue weights and adipocyte areas showed a significant decrease with NHDC and GNHDC supplementation. However, visceral fats have been targeted more than subcutaneous fats in terms of diabetes and obesity [51,52]. Visceral adipose tissue is related to type 2 diabetes mellitus [53] and dyslipidemia [54,55]. There is a link between visceral adipose tissue and the liver, which involves glucose metabolism [56]. In the present study, visceral adipose tissues and liver weight tended to decrease in the GNHDC group as compared to the Ctrl group, but this was not significant. This result suggests that NHDC and GNHDC improve lipogenesis, adipogenesis, β-oxidation, and fat browning while having little effects on regulating blood glucose levels.

A further in vitro study investigated the PI3K/AKT/mTOR pathway to understand the mechanism of the anti-obesity effects identified in the in vivo study. The activation of the PI3K/AKT/mTOR pathway increases cell proliferation and lipid synthesis through lipogenesis and adipogenesis [57]. The PI3K/AKT/mTOR pathway regulates lipogenesis via SREBP1C [57] as well as cell proliferation and glucose homeostasis [58]. In particular, the effects of NHDC and GNHDC on lipogenesis were more remarkable than their effects on adipogenesis in 3T3-L1 cells. Lipogenesis is defined as a process of fatty acid synthesis in the liver or adipose tissues [7]. Circulating free fatty acids are the main substrates used for the biosynthesis of long chain fatty acids and lipid accumulation. In this process, SREBP1C contributes to the synthesis of fat in adipose tissues [59]. On the other hand, the expression of mTOR is inhibited by the up-regulation of AMPK [60]. Previously, it was reported that sweeteners suppressed the PI3K/AKT/mTOR pathway and activated AMPK activation [61,62,63]. Consistently, the present study demonstrated that these sweeteners regulated this lipogenic pathway.

Several lipid mechanisms have been connected to each other by regulating fat accumulation and loss. Adipogenesis regulators are linked to fat browning [64]. For example, PPARγ is highly activated in subcutaneous adipose tissues [65], and it affects fat browning-related genes by stimulating the UCP enhancer during cell differentiation and by up-regulating adipocyte-specific UCP1 expression [66]. PGC1α is involved in a wide range of biological mechanisms such as adipogenesis, triacylglycerols metabolism, β-oxidation, and fat browning [67]. PGC1α is a binding partner of PPARγ in brown adipose tissue (BAT), and it increases PPARγ transcriptional activity to stimulate brown adipocyte differentiation. In addition, PGC1α activates UCP1 transcription and increases thermogenesis in cold environments [10,68]. Moreover, PGC1α up-regulates β-oxidation-related markers, including ACSL and CPT1α, which are crucial for the regulation of fatty acid in BAT.

A correlation between fat browning and glycosides has recently been reported. As mentioned earlier, glycosidic substances inhibited glucose reabsorption by suppressing SGLT’s glucose transport. Empagliflozin, known as an SGLT2 inhibitor, regulates fat browning-related gene expression [69] such as UCP1, cell death-inducing DNA fragmentation factor alpha-like effector A (CIDEA), PRDM16, and PGC1α. This present study also found that GNHDC significantly up-regulated fat browning markers. The glycosidic structure of the sweetener activated fat browning [70], which may be one of anti-obesity mechanisms of GNHDC. Thus, NHDC and GNHDC exerted an anti-obesity effect by increasing fat browning through the regulation of AMPK targets such as PGC-1 α, PRDM16, and UCP1.

Interestingly, GNHDC exerted a better fat reduction effect than NHDC did. Previous studies have reported that glycosides of the flavonoid group could suppress lipid accumulation and related gene expressions in in vivo animal studies. Quercetin from flavonol and its glycoside molecules down-regulated adipogenesis and up-regulated browning markers through AMPK activation. Body weight gain and lipid accumulation were decreased in adipose tissues by the glycoside groups [71]. That study demonstrated that the differences in efficiency were due to bioavailability or conjugated forms. The bioavailability of glycosides are important for their metabolites [72]. From this perspective, the major metabolites of NHDC are hesperetin dihydochalcone and hesperetin [73]; however, for GNHDC, it remains unclear. Flavonoid glycosides from seabuckthorn leaves reduced the size of the lipid droplets in white adipose tissue [74], and kaempferol glycosides down-regulated the mRNA expression levels of *Pparγ* and *Srebp1c* [29]. It is noteworthy that the present study showed a higher efficiency of glycosidic sweetener on lipogenesis, and that adipogenesis occurred in vivo.

Regarding glycoside absorption, the structure of glycosidic compounds affected glucose uptake via SGLT and the glucose transporter (GLUT) in human intestinal epithelial Caco-2 cells [75]. This study illustrated that the molecular structures and the number of sugars added to the flavonoid compound affected its absorption. Several organs such as the liver, intestines, and kidney are involved in the metabolic processing of glycosides [72]. For these reasons, metabolic processes may explain their higher efficiency in animal studies than in in vitro studies. However, the underlying mechanism of GNHDC has not been elucidated. The pharmacokinetic parameters of the bioavailability and the metabolites of the glycosidic sweetener need to be investigated in future studies.

## 5. Conclusions

NHDC and GNHDC caused a reduction of fat and lipid accumulation in db/db mice and 3T3-L1 cells. Both sweeteners regulated the expression of genes involved in fatty acid uptake, lipogenesis, adipogenesis, β-oxidation, and fat browning in vivo. The sweeteners down-regulated genes involved in lipogenesis and adipogenesis, proinflammatory cytokines, and the PI3K/AKT/mTOR pathway, and they up-regulated AMPK phosphorylation in vitro. These results were more significant with GNHDC. It is necessary to further examine the mechanism of glycosides in terms of regulating glucose and lipid metabolism. Thus, NHDC and its glycosides, GNHDC, have the promising potential of reducing subcutaneous fat and lipid accumulation through the modulation of the PI3K/AKT/mTOR and AMPK pathway-related molecular markers.

## Figures and Tables

**Table 1:** AIN-93G diet composition.

Ingredient	AIN 93G
	gm
Casein, lactic	200
L-Cystine	3
Corn Starch	397
Sucrose	100
Dextrose	132
Cellulose	50
Soybean Oil	70
t-Butylhydroquinone	0.014
AIN-93G Mineral Mix	35
AIN-93 Vitamin Mix	10
Choline Bitartrate	2.5
Total	1000

**Table 2:** Primer sequences for quantitative real-time PCR.

Name/GeneID		Forward Primer (5′ to 3′)	Reverse Primer (5′ to 3′)
*Acox1* (Acyl-CoA oxidase 1)	11430	TTGGAAACCACTGCCACATA	AGGCATGTAACCCGTAGCAC
*Acsl1* (Acyl-CoA synthetase long-chain family member 1)	14081	TGCCAGAGCTGATTGACATTC	GGCATACCAGAAGGTGGTGAG
*Cd36* (Cluster of differentiation 36)	12491	GTGCTCTCCCTTGATTCTGC	TGAGAATGCCTCCAAACACA
*C/ebpα* (CCAAT/enhancer binding protein alpha)	12606	CCAAGAAGTCGGTGGACAAGA	CGGTCATTGTCACTGGTCAACT
*Cpt1α* (Carnitine palmitoyltransferase 1 alpha)	12894	AACCCAGTGCCTTAACGATG	GAACTGGTGGCCAATGAGAT
*Fas* (Fatty acid synthase)	14104	TGTGAGTGGTTCAGAGGCAT	TTCTGTAGTGCCAGCAAGCT
*Il-1β* (Interleukin 1 beta)	16176	ATGGCAACTGTTCCTGAACTCAACT	CAGGACAGGTATAGATTCTTTCCTTT
*Lpl* (Lipoprotein lipase)	16956	GAGTTTGACCGCCTTCCG	TCCCGTTACCGTCCATCC
*Mcp1* (Monocyte chemoattractant protein 1)	17224	CTTCTGGGCCTGCTGTTCA	CCAGCCTACTCATTGGGATCA
*Pgc1α* (Peroxisome proliferator-activated receptor gamma coactivator 1 alpha)	19017	TCGAGCTGTACTTTTGTGGA	TCATACTTGCTCTTGGTGGA
*Pparγ* (Peroxisome proliferator-activated receptor gamma)	19016	GAGCACTTCACAAGAAATTACC	GAACTCCATAGTGGAAGCCT
*Prdm16* (Positive regulatory domain 16)	70673	AGATGAACCAGGCATCCACT	TCTACGTCCTCTGGCTTTGC
*Srebp1c* (Sterol regulatory element-binding protein 1)	20787	TAGAGCATATCCCCCAGGTG	GGTACGGGCCACAAGAAGTA
*Tnfα* (Tumor necrosis factor alpha)	21926	ATGAGAAGTTCCCAAATGGC	CTCCACTTGGTGGTTTGCTA
*Ucp1* (Uncoupling protein 1)	22227	CCAAGCCAGGATGGTGAAC	CCAGCGGGAAGGTGATGATA
*Glut4* *(Glucose transporter type 4)*	20528	TGTTCAATCACCTGGTTGCG	CTTGGCTCCCTTCAGTTTGG
*Gapdh* (Glyceraldehyde 3-phosphate dehydrogenase)	14433	AACTTTGGCATTGTGGAAGG	TGTGAGGGAGATGCTCAGTG

**Table 3:** Effects on body weight, tissue weight, plasma lipid profile, and cytokines in db/db mice.

	Ctrl	NHDC	GNHDC	*p*-Value
Final body weight (g)	37.75 ± 1.02 ^a^	35.23 ± 0.64 ^a^	35.32 ± 1.26 ^a^	0.1551
Body weight gain (g)	9.80 ± 0.67 ^a^	8.01 ± 0.69 ^a^	6.95 ± 0.79 ^b^	0.0314
Food intake (g/day)	6.25 ± 0.20 ^a^	5.90 ± 0.20 ^a^	6.00 ± 0.27 ^a^	0.5340
Water intake (mL/day)	18.31 ± 1.47 ^a^	14.68 ± 1.65 ^a^	14.92 ± 1.68 ^a^	0.2216
Liver (g)	2.31 ± 0.05 ^a^	2.13 ± 0.09 ^a^	2.08 ± 0.12 ^a^	0.1874
Subcutaneous adipose tissues (g)	2.46 ± 0.47 ^a^	1.27 ± 0.16 ^b^	1.49 ± 0.19 ^b^	0.0247
Visceral adipose tissues (g)	1.22 ± 0.07 ^a^	1.22 ± 0.07 ^a^	1.12 ± 0.04 ^a^	0.4228
Total adipose tissues (g)	5.66 ± 0.49 ^a^	4.42 ± 0.20 ^b^	4.57 ± 0.24 ^b^	0.0299
Total cholesterol (mg/dL)	159.39 ± 3.60 ^a^	158.81 ± 2.57 ^a^	146.17 ± 4.03 ^b^	0.0193
LDL-cholesterol (mg/dL)	76.50 ± 4.80 ^a^	73.47 ± 2.06 ^a^	61.29 ± 6.37 ^a^	0.0766
HDL-cholesterol (mg/dL)	53.54 ± 3.02 ^a^	53.82 ± 2.10 ^a^	54.77 ± 5.21 ^a^	0.9205
Triacylglycerols (mg/dL)	146.78 ± 3.77 ^a^	157.61 ± 7.09 ^a^	146.91 ± 11.81 ^a^	0.5736
NEFA (mEq/L)	0.73 ± 0.04 ^a^	0.63 ± 0.02 ^a^	0.61 ± 0.04 ^b^	0.0444
Leptin (ng/dL)	30.05 ± 1.24 ^a^	31.27 ± 1.74 ^a^	25.67 ± 3.37 ^a^	0.2161
Adiponectin (ng/dL)	0.54 ± 0.02 ^ab^	0.50 ± 0.02 ^a^	0.62 ± 0.04 ^b^	0.0377
Insulin (ng/dL)	1.77 ± 0.28 ^a^	2.95 ± 0.36 ^b^	1.42 ± 0.24 ^a^	0.0035
HOMA-IR	41.28 ± 21.05 ^ab^	58.86 ± 20.98 ^a^	29.83 ± 19.39 ^b^	0.0206

All data are presented in mean ± SEM (*n* = 9 per group) and analyzed by one-way ANOVA with a Newman–Keuls post hoc test. The *p*-values for one-way ANOVA are presented. NEFA, non-esterified fatty acid; HOMA-IR, Homeostatic Model Assessment for Insulin Resistance; Ctrl, control; NHDC, neohesperidin dihydrochalcone; GNHDC, NHDC-O-glycoside. The same letter indicates no significant differences (*p* > 0.05) and different letters indicate significant differences (*p* < 0.05, ANOVA).

## Data Availability

Most of the data can be found in the manuscript, and further data are available on reasonable request from the corresponding author.

## Sample Availability

Samples of the compounds are not available from the authors.
